#  Changes in Iron Status Are Related to Changes in Brain Activity and Behavior in Rwandan Female University Students: Results from a Randomized Controlled Efficacy Trial Involving Iron-Biofortified Beans

**DOI:** 10.1093/jn/nxy265

**Published:** 2019-03-30

**Authors:** Michael J Wenger, Stephanie E Rhoten, Laura E Murray-Kolb, Samuel P Scott, Erick Boy, Jean-Bosco Gahutu, Jere D Haas

**Affiliations:** 1 Department of Psychology and Cellular and Behavioral Neurobiology, The University of Oklahoma, Norman, OK; 2 Division of Nutritional Sciences, Cornell University, Ithaca, NY; 3 Department of Nutritional Sciences, The Pennsylvania State University, University Park, PA; 4 HarvestPlus, International Food Policy Research Institute, Washington, DC; 5 University of Rwanda, College of Medicine and Health Sciences, Huye, Rwanda

**Keywords:** biofortification, iron, cognition, brain, women of reproductive age

## Abstract

**Background:**

Evidence suggests that iron deficiency (ID) affects cognitive performance, as measured in behavior. Although such effects must be mediated by changes in the brain, very few studies have included measures of brain activity to assess this relation.

**Objective:**

We tested the hypothesis that provision of iron-biofortified beans would result in improvements in measures of iron status, brain dynamics, and behavior.

**Methods:**

A double-blind, randomized, intervention study was conducted in 55 women aged 18–27 y with low iron status (serum ferritin <20 µg/L). Women were randomly assigned to consume iron-biofortified (86.1 ppm iron) or comparison beans (50.1 ppm iron) daily for 18 wk. Iron status was assessed by hemoglobin, ferritin, transferrin receptor, and body iron; cognitive performance with 5 computerized tasks; and brain dynamics by concurrent electroencephalography (EEG). All measures were taken at baseline and endline.

**Results:**

The groups did not differ on any measures at baseline. Intention-to-treat analyses revealed significant (all *P* < 0.05) improvements in hemoglobin (partial effect size attributable to the independent variable, η^2^ = 0.16), ferritin (η^2^ = 0.17), and body iron (η^2^ = 0.10), speed of responding in attentional and mnemonic tasks (η^2^ = 0.04-0.29), sensitivity and efficiency of memory retrieval (η^2^ = 0.12-0.55), and measures of EEG amplitude and spectral power (η^2^ = 0.08 to 0.49). Mediation models provided evidence in support of the hypothesis that changes in iron status produce changes in behavior by way of changes in brain activity.

**Conclusions:**

Behavioral performance and brain activity, as measured by EEG, are sensitive to iron status, and the consumption of iron-biofortified beans for 18 wk resulted in improvements in measures of both, relative to what was obtained with a comparison bean, in a sample of female university students. Furthermore, the results support the conclusion that changes in brain activity resulting from consumption of biofortified beans mediate the relations between changes in iron biomarkers and changes in cognition. Clinical trial registry: ClinicalTrials.gov Reg No. NCT01594359.

## Introduction

Iron deficiency (ID) is the most prevalent nutritional deficiency worldwide and anemia its most common clinical consequence (1). There has been substantial interest in documenting the efficacy of iron interventions including supplementation, commercial fortification (2), point-of-use fortification (3), and biofortification (4). There is accumulating evidence that these interventions are successful at addressing ID and iron deficiency anemia at the level of biomarkers for iron. There is also evidence that improvements in iron status are accompanied by improvements in physical and cognitive performance (5–7).

One critical gap in the human literature is with respect to the relation between changes in systemic iron and changes in functional outcomes. In all conceivable cases, to produce a change in a cognitive or affective outcome there needs to be a change in neural function. The 4 possible iron-dependent mechanisms for changes in neural function include myelination, neurotransmitter synthesis and regulation, neurogenesis and synaptogenesis, and energy regulation and expenditure (8, 9). The time-course for each of these mechanisms varies widely, with the most likely candidates for change in adults over a 3–6 mo period (the duration of many interventions, including supplementation (6)) being regulation of neurotransmitter synthesis and energy expenditure. There is substantial literature on the effects of ID on brain structure and function (9, 10), and growing literature on the links between iron status and cognitive functioning (5, 11–13). However, the data on the extent to which changes in brain state are intermediary between changes in systemic iron levels and changes in behavioral measures of cognitive functioning are sparse (e.g., 13, 14). Thus, to understand how nutrient-specific interventions produce changes in any functional outcome, it is necessary to test the specific hypothesis that these interventions produce concurrent changes in measures of brain activity and behavior.

The present study tested the hypothesis that consumption of additional iron from a biofortified bean (BFB) would result in (a) improvements in a range of cognitive functions and (b) concomitant changes in electroencephalographic (EEG) measures of brain activity. In addition, the data were used to assess the extent to which changes in brain activity mediate the relation between changes in systemic iron status and changes in behavior.

## Materials and Methods

### Subjects

**FIGURE 1:**
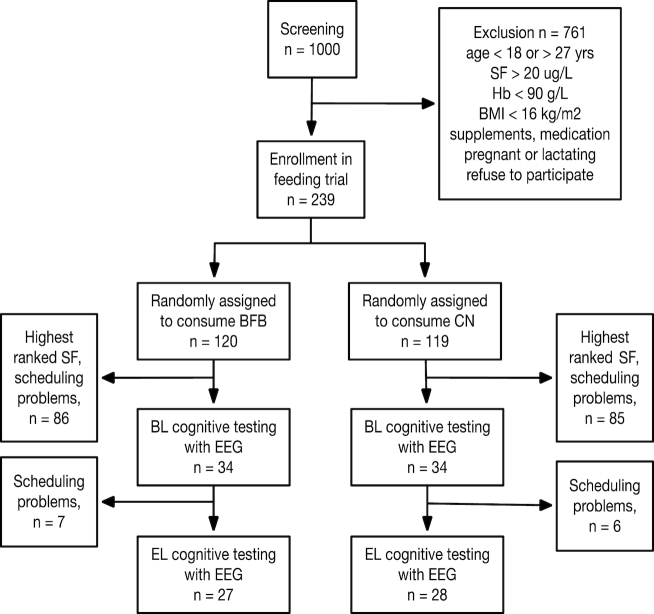
Flow diagram for screening, selection, and testing. BFB, biofortified beans; BL, baseline; BMI, body mass index; CN, comparison beans; EL, endline; EEG, electroencephalography; Hb, hemoglobin; SF, serum ferritin.

Subjects were a subset of women of reproductive age attending the University of Rwanda in Butare (altitude 1768 m) who were originally recruited for a parent study of the efficacy of consuming iron-BFB (4). **Figure 1** summarizes the process of recruitment, random assignment, and testing. Exclusion criteria were the following: age outside the range of 18–27 y, pregnant or lactating, BMI < 16 kg/m^2^, hemoglobin (Hb) < 90 g/L, serum ferritin (SF) > 20 µg/L, any major medical conditions, any use of vitamin and iron-containing supplements, recent blood donation, or any use of long-term medications other than oral contraceptives. The Hb and SF cutoffs were women who were severely anemic or who were iron-sufficient. After screening 1000 women 2 mo before the start of the feeding trial, 239 were eligible and enrolled in the parent study (see (4)).

The minimum sample size estimated to allow for 80% power and α = 0.05 for differences in the amplitude of event-related potentials (ERPs), based on our as-yet-unpublished data on another study incorporating EEG, as well as EEG data from Otero et al. (13), was 30 per group. Consequently, a subset of 68 subjects with the lowest SF levels (assessed by ranking before treatment assignment) was chosen to undergo cognitive testing with concurrent EEG, as this subgroup was thought to have the greatest potential to benefit from the intervention and, therefore, demonstrate cognitive change. Of the 68 who were tested before feeding began (at baseline, BL), 55 were re-tested at the end of the feeding trial (endline, EL). We failed to reach this sample size because we underestimated the constraints in scheduling the women at EL for the EEG and behavioral testing.

### Study design

Complete details of the study design are available in Haas et al. (4). In brief, the study was a double-blind randomized efficacy trial. Consumption occurred from 7 January to 23 May, 2013, for a total of 128 d. Women were randomly assigned to 1 of 4 color-coded groups. Two colors represented BFB (86.1 ppm iron) and 2 colors represented the comparison beans (CN, 50.1 ppm iron); 4 colors were used to ensure that all parties remained blind to treatment conditions. Beans were consumed at lunch and dinner in a cafeteria setting. The acceptable portion size, 175 g cooked weight of beans per meal per woman, was based on pilot data, and actual consumption was ad libitum with the option to return for an additional serving. All subjects consumed from the same buffet of limited choices of side dishes. Plate waste was weighed after each meal to determine the amount of beans consumed. Blood samples were obtained and cognitive tests with concurrent EEG were conducted at BL and EL.

### Laboratory analysis

Whole blood samples were collected between 0900 and 1100 in heparin tubes from an antecubital vein at BL and EL by a trained phlebotomist and analyzed at the Rwanda Military Hospital within 6 h of collection for complete blood count, including Hb. A second tube without anticoagulant was collected and centrifuged to separate serum, which was frozen and later analyzed for SF, soluble transferrin receptor (sTfR), C-reactive protein, and α-1-acid glycoprotein by the VitMin Laboratory after a sandwich ELISA procedure (15). Total body iron stores were estimated as the log of the ratio of sTfR and SF, according to Cook's equation (16). Laboratory samples were tested in batch by a senior technician, and instruments were calibrated daily based on standardized procedures. A constant 6 g/L was subtracted from Hb values to account for altitude. Anemia was defined as Hb < 120 g/L, iron deficiency as SF < 15 ug/L, sTfR > 8.3 mg/L or total body iron < 0 mg/kg, and inflammation as C-reactive protein > 5.0 mg/L or α-1-acid glycoprotein > 1.0 mg/L.

### Cognitive tasks

All subjects were tested in a 60–90 min session once within 3 wk after bean consumption started and once in the final 3 wk of the study. Task instructions were given in the local language (Kinyarwanda) by trained research technicians. Subjects were seated at an unconstrained distance ∼70 cm from the screen of the testing computer.

Five measures of attentional and mnemonic functioning were used. We selected measures that recruit brain systems that have been documented, in either the human or animal literatures, to have some dependency on iron status. Also, we selected tasks that have been used extensively in the human experimental literature, to allow for comparisons with other studies. All tasks were computer-based and included standardized explanatory instructions and practice trials.

The tasks were developed and programmed by MJW using Eprime (Psychology Software Tools); programs and stimuli are freely available on request. The tasks were presented on Windows-based laptop computers with 36 cm (diagonal) displays, running at 2.5 GHz, with at least 4 Gb of RAM and at least 320 Gb of hard disk storage. Stimulus onsets were synchronized to the vertical refresh rate of the monitor and keyboard responses were timed to ±1 ms. Stimuli for all of the tasks were either grayscale images or white characters on a black background. Example stimuli for each of the tasks and the order in which the tasks were presented are shown in **Supplemental Figure 1**. The dependent variables from each of the tasks, including the EEG variables are presented in **Table 1**, along with the direction of change associated with improvement. Complete procedural details are in the online Supplemental material.

**FIGURE 2:**
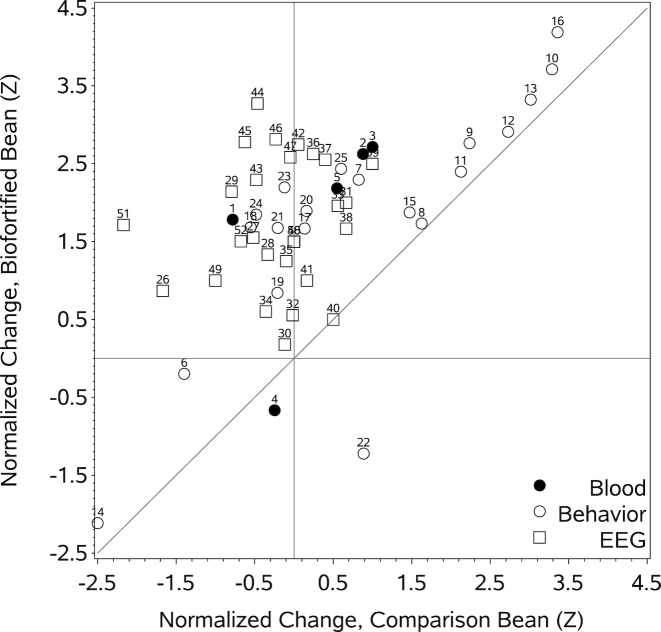
Normalized (Z-unit) change scores for each of the 3 classes of dependent variables (blood, behavior, and EEG) for the participants in the biofortified bean treatment condition relative to those in the comparison bean treatment condition. Numbers above each of the plot symbols refer to the variable numbers in Supplemental Table 1.

**TABLE 1:** Definitions of each of the dependent variables in the cognitive tasks, including the features of the concurrent EEG collected in all tasks^1^

Task	Variable	Direction of improvement^2^	Definition
SRT	RT	↓	Median RT for correct responses
GNG	RT	↓	Median RT for correct responses
ANT	RT, 0 cues	↓	Median RT for correct responses to stimuli with 0 cues (RT_0_)
	RT, 2 cues	↓	Median RT for correct responses to stimuli with 2 cues (RT_2_)
	RT, alerting	↑	RT_0_ - RT_2_
	RT, center cue	↓	Median RT for correct responses to stimuli with center cues (RT_C_)
	RT, spatial cues	↓	Median RT for correct responses to stimuli with spatial cues (RT_S_)
	RT, orienting	↑	RT_C_ - RT_S_
	RT, consistent flankers	↓	Median RT for correct responses to stimuli with consistent flankers (RT_n_)
	RT, inconsistent flankers	↓	Median RT for correct responses to stimuli with inconsistent flankers (RT_i_)
	RT, conflict	↑	RT_n_ - RT_i_
CRT	RT, new items	↓	Median RT for correct responses to new stimuli presented with all 4 quadrants visible
	RT, old items	↓	Median RT for correct responses to old stimuli presented with all 4 quadrants visible
	Sensitivity (d')	↑	Sensitivity to the presence of old items with all found quadrants visible, calculated as d' = Z^−1^(hit rate) − Z^−1^(false alarm rate)
	Bias (c)	↓↑^3^	Propensity to report an item with all 4 quadrants visible as old, calculated as c = [Z^−1^(hit rate) + Z^−1^(false alarm rate)]
	PCC	↑	Percentage change in capacity, based on the proportionality estimate β^∧^ obtained from the proportional hazards model, = × 100
SMS	RT, intercept, new items	↓	Intercept from equation regressing RT for new items on number of items
	RT, intercept, old items	↓	Intercept from equation regressing RT for old items on number of items
	RT, slope, new items	↓	Slope from equation regressing RT for new items on number of items
	RT, slope, old items	↓	Slope from equation regressing RT for old items on number of items
EEG	Peak amplitude, P1	↑	Peak amplitude of the first positive-going ERP, indexing initial perceptual encoding
	Peak amplitude, N1	↓	Peak amplitude of the first negative-going ERP, indexing object-level representation
	Peak amplitude, P2	↑	Peak amplitude of the second positive-going ERP indexing semantic processing
	α-band power, normalized, change from baseline	↑	Normalized change in power in the 8–15 Hz band, reflecting relaxed, focused attention and responses to changes in task difficulty
	γ-band power, normalized, change from baseline	↑	Normalized change in power in the 30–90 Hz band, reflecting effortful sustained attention

^1^ANT, attentional network task; CRT, cued recognition task; EEG, electroencephalography; ERP, event-related potential; GNG, go/no-go task; PCC, percentage change in capacity; RT, reaction time; SMS, Sternberg memory search task; SRT, simple reaction time task.

^2^Indicates which direction (higher or lower) indicates better performance.

^3^Optimal or unbiased performance is near zero; deviation from zero in either direction indicates either a conservative (<0) or liberal (>0) response bias.

The simple reaction time (SRT) task provides an estimate of the speed of the simplest possible behavioral response to a visual stimulus. The go/no-go (GNG) task provides an estimate of the efficiency of sustained attention and the speed of attentional capture in the absence of a need to filter competing information. The attentional network task (ANT) provides an estimate of the effectiveness of 3 components of attention: alerting (low-level attentional capture), orienting (mid-level spatial selective attention), and conflict [high-level selection (17)]. The Sternberg memory search (SMS) task (18) estimates the speed and accuracy with which immediate visual memory can be searched. The cued recognition task (CRT) follows a modified (19) version of a classic visual recognition memory task (20) that estimates the speed, accuracy, and efficiency of recognition based on short-duration visual memory.

### EEG data acquisition

Concurrent EEG were acquired using a 64 Ag/AgCl electrode cap (BrainCap, BrainProducts), connected to a 64-channel DC-powered amplifier (BrainAmp, BrainProducts). EEG, as measured at the scalp, reflects the coordinated activity of spatially adjacent populations of similarly oriented neurons, propagated through the brain, skull, and skin (21). Their activity corresponds to the computational operations that are needed to allow stimulus to be transformed into a response. To the extent that these populations of neurons are efficiently carrying out these computations, the amplitude and power of their conjoint activity will be higher, relative to any iron-dependent impairments (e.g., neurotransmitter signaling or oxygen transport). Data were digitized at 1 KHz during acquisition and downsampled to 250 Hz for analyses. Impedances were maintained at <5 kΩ during acquisition. Preprocessing for statistical analysis is described in the online Supplemental material.

### Ethics

All subjects provided written informed consent. Approval of the research was granted by the Institutional Review Boards of Cornell University, The University of Oklahoma, The Pennsylvania State University, and the Rwanda National Ethics Committee. The study was registered with ClinicalTrials.gov, #NCT01594359.

### Statistical analyses

All analyses were performed using SAS 9.4 for Linux (2017, SAS Institute). Differences in demographic and BL iron status biomarker values were assessed using 2-tailed *t* tests. Changes in blood, behavioral, and EEG variables were assessed using 2 classes of analyses. The first was intention-to-treat analyses in which the EL value of each variable was modeled as a function of the treatment condition (BFB, CN), using the BL value as a covariate. The second class was applied to the changes in the behavioral and EEG data across time, to assess the plausibility of iron repletion as a source of the observed changes. This involved regressing change in the behavioral and EEG variables onto change in the battery of iron status biomarkers, testing the hypothesis that women who experienced a greater improvement in iron status would show greater improvement in their behavioral and brain measures. The set of candidate models included a “null” model (intercept only); a full model, in which all allowable independent predictors (iron biomarkers) were included; and a model whose form was determined by step-wise model selection procedures to minimize the number of parameters while maximizing *R^2^*. We selected a best model using the criteria that the model had to provide a better account than the null model, than any of the competing models [as assessed using the Akaike Information Criterion (22)], and account for at least 10% of the variance.

The intention-to-treat and secondary analyses of the EEG variables included a preliminary step to identify, for each subject, the electrode that collected activity that was most responsive to the experimental manipulations. Five features of the EEG were selected for analyses: peak amplitude (μV) and time to peak amplitude (ms) for 3 ERP features—P1, N1, and P2—along with mean normalized spectral power in the α- and γ-bands (see Table 1 and **Supplemental Figure 2**). Before the intention-to-treat and secondary analyses, the dependent measures were regressed onto treatment condition (BFB, CN), time of assessment (EL, BL), and the treatment-by-time interaction separately for each electrode in each of 5 regions on the scalp (frontal, central, left and right temporo-parietal, and occipital). The electrode for which this regression model accounted for the greatest *R^2^*, conditional on that being ≥10%, was then selected to provide the data for the intention-to-treat analyses and secondary analyses, which were of the same form as used for the behavioral variables.

The third class of analyses involved estimating a set of mediation models incorporating effect modification (23) to represent the hypothesis that changes in brain activity mediate the relation between changes in iron status and changes in behavior. These analyses were performed on 3 composite (*Z*-transformed) behavioral variables: change in low-level attentional capture, change in high-level attentional selection, and change in efficiency of memory search. The attentional capture variable was formed by averaging the *Z-*transformed change in reaction time (RT) in the SRT and GNG, along with the change in RT in the 2-cue condition in the ANT. The attentional selection variable was formed by averaging the *Z*-transformed change in RT in the spatial cue and inconsistent flanker conditions of the ANT. The memory efficiency variable was formed by averaging the *Z*-transformed change in slope and intercept for the SMS and the variation in percentage change in capacity (PCC) in the CRT, separately for new and old items. The possible mediating and moderating variables were the normalized (*Z*-transformed) change in N1 amplitude and change in α- and γ-power from baseline. The possible predictors were the normalized (*Z-*transformed) change in Hb and SF.

These variables were used to fit candidate mediation models with effect modifiers for each behavioral composite score, using all possible combinations of the EEG composite scores as both mediators and effect modifiers. Along with these models, 2 additional models, a “direct effect only” model and a “scrambled” model, were fit to the data for each composite behavioral score. The “direct effect only” model did not include any mediators or effect modifiers, and the “scrambled” model was a model created by rearranging the order of the effects in the best-fitting model. The “best” model met the following criteria: (a) the overall *F*-statistic for the model had to be statistically significant; (b) all component *R^2^* values needed to be ≥0.10; (c) all values for model parameters (excluding the intercept) needed to be significantly different from 0; and, (d) based on *R^2^* and Akaike Information Criterion values, the “best” model had to outperform all alternative models, including the “direct effect only” and “scrambled” models.

## Results

**Table 2** presents the baseline demographics and prevalence for anemia, iron deficiency, and inflammation. No significant differences between the 2 treatment conditions were observed for any of these baseline measures. **Supplemental Table 1** presents the descriptive statistics for BL, EL, and change values (EL–BL) of all of the dependent variables for subjects in both conditions. **Figure 2** plots the normalized change values (mean change in each condition divided by the pooled SD, scaled so that positive values indicate improvement) for the BFB against the CN conditions. Here it can be seen that the majority of the variables lie above the line of unity, indicating greater improvement for those who consumed the BFB relative to those who consumed the comparison beans.

**TABLE 2:** Baseline demographic characteristics and prevalence of anemia and iron deficiency at baseline^1^

	CN (*N* = 28)	BFB (*N* = 27)
Age, y	22.4 (0.3)	22.9 (0.3)
BMI, kg/m^2^	22.4 (0.5)	22.9 (0.6)
Anemia, Hb < 12 g/dL, *n* (%)	8 (29)	12 (44)
Iron deficiency, *n* (%)		
SF < 15 μg/L	26 (93)	21 (78)
sTfR > 8.3 mg/L	10 (36)	8 (30)
BdFe < 0 mg/kg^2^	17 (61)	14 (52)
Iron deficiency anemia, Hb < 12 g/dL and SF < 15 μg/L	8 (29)	11 (41)
Iron deficiency without anemia, Hb ≥ 12 g/dL and SF < 15 μg/L	18 (64)	10 (37)
Inflammation, AGP > 1.0 g/L or CRP > 5.0 mg/L, *n* (%)	1 (4)	2 (7)

^1^Entries are either means (standard errors) or *N*s (percentages). No significant differences were observed between the groups on any of these baseline measures. AGP, α-1 acid glycoprotein; BdFe, body iron; BFB, biofortified beans; BMI, body mass index; CN, comparison beans; CRP, C-reactive protein; Hb, hemoglobin; SF, serum ferritin; sTfR, soluble transferrin receptor

### Intention-to-treat analyses: blood variables

**Table 3** summarizes the results of the intention-to-treat analyses. Each of the iron status variables showed a significant effect of treatment condition, controlling for BL, except for sTfR. These results are consistent with those obtained in the analyses of data from all subjects (*n* = 150) who participated in the cognitive testing (12), and for all subjects (*n* = 239) in the feeding trial (4), where significant or marginally significant effects associated with treatment condition were obtained for all iron status variables except sTfR.

**TABLE 3:** Intention-to-treat analyses of the blood, behavioral, and EEG variables, examining the effect of treatment condition on EL measures, controlling for BL^1^

		Effect of BL value			Effect of treatment condition		
Outcome/task	EL variable	*F*	MSE	Partial η^2^	*F*	MSE	Partial η^2^
Blood	Hb	257.08^&^	0.34	0.83	10.25^%^	0.34	0.16
	SF	37.10^&^	33.59	0.42	10.20^%^	33.59	0.17
	log_10_(SF)	53.16^&^	0.02	0.51	8.72^%^	0.02	0.15
	sTfR	165.41^&^	3.80	0.76	0.95	3.80	0.02
	BdFe	125.24^&^	2.12	0.71	5.43*	2.12	0.10
Behavioral variables							
SRT	Median RT	60.43^&^	1407	0.54	6.18*	1407	0.11
GNG	Median RT	36.82^&^	1402	0.44	1.86	1402	0.02
ANT	Alerting score	3.47^+^	2739	0.02	0.33	2739	0.00
	RT, 0 cues	61.67^&^	3687	0.29	0.06	3867	0.00
	RT, 2 cues	53.22^&^	3971	0.26	0.02	3971	0.00
	Orienting score	0.06	2124	0.00	4.82*	2124	0.04
	RT, center cues	60.03^&^	3520	0.28	0.30	3520	0.00
	RT, spatial cues	76.04^&^	2952	0.33	1.96	2952	0.01
	Conflict score	10.04^%^	2029	0.06	0.11	2029	0.00
	RT, consistent flankers	82.11^&^	3352	0.35	0.10	3352	0.00
	RT, inconsistent flankers	77.86^&^	3609	0.34	14.26^&^	3609	0.09
SMS	RT intercept, new items	2.21	13,105	0.04	13.40^&^	13,105	0.22
	RT intercept, old items	13.36^&^	13,852	0.22	21.69^&^	13,852	0.29
	RT slope, new items	0.53	358	0.01	11.47^%^	358	0.19
	RT slope, old items	0.05	814	0.00	9.79^%^	814	0.17
CRT	Sensitivity, 4-cue trials	9.28^%^	0.20	0.15	63.45^&^	0.20	0.55
	Criterion, 4-cue trials	3.64^+^	0.10	0.07	10.55^%^	0.10	0.17
	RT, 4-cue trials, new items	15.56^&^	10,230	0.24	28.49^&^	10,230	0.37
	RT, 4-cue trials, old items	18.22^&^	10,748	0.27	9.42^%^	10,748	0.16
	Percentage change in capacity	0.86	2349	0.02	22.88^&^	2349	0.32
EEG variables							
SRT	N1 amplitude, central	1.49	1.36	.08	17.23^&^	1.36	0.49
	P2 amplitude, central	0.73	0.05	0.11	2.75^+^	0.05	0.27
	α-power, central	7.90^%^	0.01	0.17	10.80^%^	0.01	0.22
GNG	N1 amplitude, central	13.86^&^	4.19	0.22	3.28*	4.19	0.08
	P2 amplitude, central	11.78^%^	6.15	0.29	0.95	6.15	0.03
	α-power, central	7.47*	0.001	0.20	0.44	0.001	0.02
ANT	N1 amplitude, parietal, 0 cues	16.85^&^	1.71	0.30	4.69*	1.71	0.11
	N1 amplitude, occipital, 2 cues	33.70^&^	2.18	0.46	14.05^&^	2.18	0.27
	N1 amplitude, parietal, central cues	6.48*	1.96	0.14	3.02+	1.96	0.09
	N1 amplitude, parietal, spatial cues	21.73^&^	0.74	0.35	9.29^%^	0.74	0.19
	N1 amplitude, occipital, consistent flankers	27.10^&^	1.72	0.40	30.46^&^	1.72	0.43
	N1 amplitude, occipital, inconsistent flankers	18.00^%^	1.71	0.31	33.76^&^	1.71	0.46
	α-power, central, 2 cues	18.14^&^	0.002	0.31	3.25^+^	0.002	0.06
	α-power, central, central cues	30.13^&^	0.001	0.43	5.79*	0.001	0.13
	α-power, central, spatial cues	20.96^&^	0.001	0.34	3.16^+^	0.001	0.09
	α-power, central, consistent flankers	23.31^&^	0.001	0.37	19.01^&^	0.001	0.32
	α-power, central, inconsistent flankers	18.83^&^	0.001	0.32	22.95^&^	0.001	0.36
SMS	N1 amplitude, central, new items, set size 1	2.00	0.41	0.05	25.56^&^	0.41	0.84
	N1 amplitude, central, old items, set size 1	3.17^+^	0.84	0.08	21.71^&^	0.84	0.74
	N1 amplitude, central, new items, set size 3	1.01	1.06	0.01	29.26^&^	1.06	0.69
	N1 amplitude, central, old items, set size 3	3.71^+^	0.49	0.09	36.73^&^	0.49	0.81
	N1 amplitude, central, new items, set size 6	1.43	0.49	0.01	34.59^&^	0.49	0.82
	N1 amplitude, central, old items, set size 6	1.16	2.29	0.01	33.45^&^	2.29	0.46
	γ-power, central, old items, set size 1	55.50^&^	0.001	0.59	5.27*	0.001	0.12
	γ-power, central, old items, set size 3	9.58^%^	0.001	0.20	3.27^+^	0.001	0.07
	γ-power, central, old items, set size 6	20.80^&^	0.001	0.50	3.42^+^	0.001	0.08
CRT	N1 amplitude, central, new items, 4 cues	5.90*	1.72	0.13	41.46^&^	1.72	0.51
	N1 amplitude, central, old items, 4 cues	0.81	2.37	0.02	35.83^&^	2.37	0.46
	γ-power, central, old items, 4 cues	21.68^&^	0.001	0.34	68.08^&^	0.001	0.61

^1^ANT, attentional network task; BdFe, body iron; BL, baseline; CRT, cued recognition task; EL, endline; GNG, go/no-go task; Hb, hemoglobin; MSE, mean square error; SF, serum ferritin; SMS, Sternberg memory search task; SRT, simple reaction time task; sTfR, soluble transferrin receptor.

^+^0.05 ≤ *P* ≤ 0.10; *P < 0.05, ^%^*P* < 0.01, ^&^*P* < 0.001; Df = 1 for all *F*-scores.

### Intention-to-treat analyses: behavioral variables

Accuracy levels in the SRT, GNG, ANT, and SMS were all >0.96, suggesting ceiling effects, and therefore we only analyzed RT for correct responses. Significant effects for treatment condition were found for SRT; ANT orienting score, and for trials involving the inconsistent flanker; SMS intercept and slope; CRT sensitivity, response criterion, RT for new and old items, and percentage change in capacity (Table 3). All effects favored the BFB group (see Supplemental Table 1 for group means).

### Intention-to-treat analyses: EEG variables

Before performing the intention-to-treat analyses, regression analyses were done to identify the specific electrode to be used for the analysis of each variable. If no electrode produced data that met our criteria for analysis, then the intention-to-treat analyses were not performed for that variable. No significant results of treatment condition were obtained for any of the latency variables (Table 3). There were no significant results for the peak amplitudes of the P1 component. However, for the peak amplitudes of the P2 component, significant effects were observed in the SRT and GNG and, in both cases, participants who consumed the BFB had higher peak amplitudes than those who consumed the comparison beans. All other significant effects in the peak amplitude variables were obtained for the N1 component and, in all cases, the effect of treatment condition (BFB compared with CN) was an increase in the peak amplitude. All of the significant effects were obtained in central electrodes, with the exception of 2 effects that were obtained in frontal electrodes immediately adjacent to the central electrodes.

With respect to changes from power from baseline in the α- and γ-bands, significant effects from treatment conditions were obtained for α-power in the SRT, GNG, and ANT. In all cases, change in normalized α-power was higher for those consuming the BFB than for those consuming the comparison beans. In contrast, for the SMS and CRT, significant increases related to treatment condition were observed for γ-power rather than α-power, with the largest increases in power obtained for those consuming the BFB rather than the comparison beans. Like the effects in peak amplitudes, all of the significant effects for α- and γ-band power were observed in central electrodes, with the exception of 1 result that was obtained in frontal electrodes immediately adjacent to the central electrodes.

### Secondary analyses: behavioral and EEG variables

**Table 4** presents the results of regression analyses testing the hypothesis that a change in iron status is related to changes in behavioral and EEG measures. With the exception of the SRT, a “best” model was identified for at least 1 variable from each task. The majority of the change in the behavioral variables was predicted by change in SF itself, change in log_10_(SF), or change in total body iron. Larger increases in 1 of these measures were related to larger reductions in RT for GNG, ANT, and CRT, larger reductions in the intercept and slope for SMS (for both old and new items), and larger increases in sensitivity in CRT. Larger increases in Hb were related to larger liberal shifts in response bias (an increased propensity to respond “old” to old and new items) and larger increases in percentage change in capacity.

**TABLE 4:** Secondary analysis of the behavioral and EEG variables, examining the relation between change in the behavioral and EEG variables and change in blood iron markers^1^

		Change in blood marker			
Task	Change variable	Predictor	Intercept	β	*R^2^*
Behavioral variables					
GNG	Median RT	log_10_(SF)	−16	−60	0.18
ANT	RT, spatial cues	SF	−166	−4	0.11
	Conflict				
	RT, consistent flankers				
	RT, inconsistent flankers	log_10_(SF)	−96	−141	0.18
SMS	RT intercept, new items	SF	−36	−24	0.35
	RT intercept, old items	SF	55	−21	0.34
	RT slope, new items	BdFe	4	−11	0.21
	RT slope, old items	BdFe	−8	−13	0.24
CRT	Sensitivity, 4-cue trials	SF	0.32	0.03	0.11
	Criterion, 4-cue trials	Hb	−0.01	−0.20	0.12
	RT, 4-cue trials, new items	SF	−1	−13	0.19
	RT, 4-cue trials, old items	BdFe	3	−50	0.32
	Percent change in capacity	Hb	53.6	26.9	0.12
EEG variables					
SRT	α-power, central electrodes	Hb	−0.01	0.11	0.13
GNG	N1 amplitude, central electrodes	Hb	−66	−60	0.13
	α-power, central electrodes	SF	−0.12	0.03	0.20
ANT	N1 amplitude, occipital, 0 cues	Hb	−1.16	−1.28	0.15
	N1 amplitude, occipital, 2 cues	Hb	−1.55	−0.89	0.11
	N1 amplitude, occipital, center cues				
	N1 amplitude, occipital, spatial cues	BdFe	−0.92	−0.34	0.10
	N1 amplitude, occipital, consistent flankers				
	N1 amplitude, occipital, inconsistent flankers	SF	−1.06	−0.11	0.12
SMS	N1 amplitude, central, new items, set size 3	log_10_(SF)	−0.28	−6.31	0.17
	N1 amplitude, central, new items, set size 6	log_10_(SF)	0.14	−6.43	0.24
	N1 amplitude, central, old items, set size 1	log_10_(SF)	0.29	−6.57	0.24
	N1 amplitude, central, old items, set size 3	log_10_(SF)	0.03	−5.39	0.19
	N1 amplitude, central, old items, set size 6	log_10_(SF)	−0.32	−4.94	0.14
	α-power, occipital, new items, set size 1	log_10_(SF)	−0.03	0.14	0.17
	α-power, frontal, new items, set size 3	SF	−0.01	0.01	0.18
	α-power, occipital, new items, set size 6	SF	−0.01	0.01	0.19
	α-power, occipital, old items, set size 1	SF	−0.02	0.01	0.20
	α-power, frontal, old items, set size 6	SF	−0.01	0.01	0.18
CRT	N1 amplitude, central, new items, 4 cues	Hb	1.08	−1.13	0.11
	N1 amplitude, central, old items, 4 cues	Hb	−0.31	−1.75	0.17
	α-power, central, new items, 4 cues	Hb	0.01	0.02	0.10
	α-power, central, old items, 4 cues	Hb	0.01	0.03	0.12

^1^Blank entries, or absence of a variable, indicate that no acceptable model was identified for that variable/analysis. All reported β-values were significantly different from 0. ANT, attentional network task; BdFe, body iron; BL, baseline; CRT, cued recognition task; EL, endline; GNG, go/no-go task; Hb, hemoglobin; SF, serum ferritin; SMS, Sternberg memory search task; SRT, simple reaction time task.

A “best” model was identified for at least 1 EEG variable in each task. Change in some function of SF was the most common predictor of change in EEG variables. All of the effects in the analyses of the ERPs were obtained for the N1 component, with all of these being localized in central electrodes, except for 1 effect in frontal electrodes immediately adjacent to the central electrodes. All of the effects in the analyses of spectral power were obtained for changes in α-band power. Larger increases in SF were related to larger increases in N1 amplitudes in the ANT (spatial cues and inconsistent flankers) and SMS, and larger increases in α-band power in GNG, SMS, and CRT. Larger increases in Hb were related to larger increases in N1 amplitude in the ANT (spatial cues and inconsistent flankers) and the SMS, and larger increases in α-band power in the GNG, SMS, and CRT.

### Mediation models

The parameters and measures of descriptive adequacy (*R^2^*) for the best-performing models for each of the composite variables are presented in **Table 5**. In addition, estimates of the total effect of the predictors and mediators, estimates of the indirect effect (per 23) of the mediators, and the ratio of the indirect to the total effect are presented. In all cases, models with mediating effects provided a better account for the data than did models that contained only direct effects, and in all cases the best-form of the mediation model was that in which change in SF was the primary predictor and change in Hb was a covariate.

**TABLE 5:** Parameters and effect sizes for the models for the mediating effects of changes in brain activity on the relation between changes in measures of blood iron and measures of cognitive performance^1^

Mediators							Primary outcome							Effect sizes		
Variable	Pred	*F*	*Int*	β	*t*	*R^2^*	Variable	Pred	*F*	*Int*	β	*t*	*R^2^*	Total	Indirect	Ratio
Model for attentional capture																
Capt α	SF	9.99^&^	0.31	0.40	4.81^&^	0.31	Capt RT	SF	8.08^&^	−0.15	0.48	2.69^%^	0.42	0.61	0.48	0.79
	Hb			0.24	2.13*			Hb			0.22	2.14*				
Capt N1	SF	6.45^%^	0.07	0.22	2.23*	0.22		Capt α			0.27	2.05*				
	Hb			0.41	2.72^%^			Capt N1			0.17	1.85^+^				
Model for attentional selection																
Seln α	SF	11.01^&^	−0.19	0.37	2.99^%^	0.31	Seln RT	SF	8.14^&^	−0.07	0.36	2.24*	0.41	0.57	0.36	0.63
	Hb			0.24	2.48*			Hb			0.19	1.92^+^				
Seln N1	SF	8.27^&^	0.18	0.34	2.29*	0.25		Seln α			0.39	2.41*				
	Hb			0.28	2.46*			Seln N1			0.19	2.06*				
Model for ME, new items																
ME new α	SF	7.84^%^	0.00	0.34	2.09*	0.25	ME new	SF	4.13^%^	0.00	0.15	1.81*	0.34	0.36	0.15	0.42
	Hb			0.32	2.48*			Hb			0.14	1.93*				
ME new γ	SF	8.60^&^	−0.05	0.76	4.07^&^	0.26		ME new α			0.10	1.72^+^				
	Hb			0.14	2.35*			ME new γ			0.15	2.09*				
ME new N1	SF	6.25^%^	0.00	0.34	1.86^+^	0.21		ME new N1			0.18	1.82*				
	Hb															
Model for ME, old items																
ME old α	SF	14.86^&^	0.05	0.93	5.45^&^	0.37	ME old	SF	8.98^&^	0.13	0.31	2.66*	0.49	0.36	0.21	0.58
	Hb			0.23	1.72^+^			Hb			0.24	3.05^%^				
ME old γ	SF	8.60^&^	−0.05	0.76	4.07^&^	0.26		ME old α			0.11	1.81^+^				
	Hb			0.17	2.05^+^			ME old γ			0.15	2.09*				
ME old N1	SF	14.40^&^	0.20	0.24	1.73^+^	0.37		ME old N1			0.18	2.11*				
	Hb			0.69	4.55^&^											

^1^In all cases, change in Hb was used as a covariate. Capt, attentional capture; Int, intercept; ME, memory efficiency; Pred, predictor; Seln, attentional selection.

^+^0.05 ≤ *P* < 0.10, *P < 0.05, ^%^*P* < 0.01, ^&^*P* < 0.001.

To understand the results presented in Table 5, consider the model for attentional capture. The first set of relations are those between the primary predictor (SF) and covariate (Hb) and the mediators (the composite variables capture α and capture N1), summarized in the first set of columns in Table 5. For both mediators, the overall model for the relation between the predictors and the mediators was significant (*F* = 9.99 and 6.45, for capture α and N1, respectively). In addition, the estimated slopes (β) for both SF and Hb were significantly different from 0 for both mediators, and the models accounted for a reasonable proportion of the variance (31% and 22%, respectively). Having established that the relations between the predictor and covariate are significant, now consider the overall model for the direct and indirect (mediating) effects of change in biomarkers on change in the behavioral measure, summarized in columns 8–14 (Primary outcome) in Table 5. Reading from left to right, we see that the overall model is significant (*F* = 8.08), the slopes for the primary predictor, the covariate, and the 2 mediating variables are also significantly different from 0, and that the overall model accounts for 42% of the variance. Finally, this approach (23) allows for bootstrapped estimates of total effect size, as well as effect sizes for the direct and indirect (mediating) effects, presented in columns 15–17 of Table 5. For attentional capture, the ratio of the indirect effect to the total effect was 0.79, which can be interpreted as the mediating effect being responsible for 79% of the total effect. The summaries of the other models can be read similarly.

Mediators for the 2 attentional composite variables were change in α-power and change in N1 amplitude. Mediators for the 2 memory composite variables were change in α-power, change in γ-power, and change in N1 amplitude. All of the models accounted for at least 34% of the variance, and the indirect effects accounted for at least 42% of the total effect of change in iron biomarkers on change in cognition. Together, these findings support the hypothesis that changes in iron biomarkers lead to changes in brain function, which then produce changes in behavior. In particular, the mediating effects of α- and γ-band power suggest that changes in both focused and effortful sustained attention lead to improvements in cognitive performance. The mediating effects of N1 amplitude suggest that changes in signal quality at a reasonably early point in the processing of stimuli lead to improvements in cognitive performance. This is because the amplitude of any EEG feature is a function of the size of the neural population that is synchronously active, which should be a function of the signals that are being transmitted at synapses in a coherent manner.

## Discussion

The present study demonstrated that consumption of iron-biofortified beans for 128 d resulted in greater positive changes in iron status, brain dynamics, and behavior, relative to consumption of comparison beans with lower iron content. Changes in behavior were found for the most challenging stimulus conditions in the ANT, along with the SMS and CRT, and no changes were found in the tasks assessing lower-level cognition (SRT, GNG, and the easiest conditions in the ANT). The changes in the EEG measures were more uniform across the tasks: effects were found for N1 amplitudes, mainly in central electrodes, with a mix of effects in α- and γ-band power. These effects, and their general topography, suggests at a minimum that the impact of iron repletion was on processing above the level of basic sensory processing. This pattern of effects was reinforced by the results of the secondary (plausibility) analysis, and the combined set of results suggests that provision of the BFB was responsible for the improvements obtained in all 3 sets of measures—blood, brain, and behavior.

All studies that probe the potential impact of manipulations of iron intake on cognitive performance are implicitly assessing the hypothesis that changes in cognitive performance result from changes in brain function. The present study had the requisite data to statistically model this implicit hypothesis by fitting mediation models with effect modifiers (23), representing the hypothesis that brain activity mediates the relation between systemic iron status and cognition at 3 levels. In the most basic—change in attentional capture—change in SF (with change in Hb as a covariate) was found to be the best predictor for the direct and indirect (mediating) effects. This is a conclusion that would not be possible on the basis of the intention-to-treat analyses and plausibility analyses alone, but is supported when the broader set of relations is considered. With respect to change in attentional selection and change in memory efficiency, change in SF was found to be the best predictor for the complete set of relations, with change in Hb as a covariate and change in α- and γ-band power and N1 amplitude mediating the relation. The current findings are similar to those we have reported for a laboratory-based study of the effects of ID on energy expenditure during a cognitive task (24). One plausible interpretation of these models is with respect to the most likely roles of SF and Hb, with SF being linked most closely to the integrity of neurotransmitter systems [dopamine in particular (25, 26)], and Hb being most closely linked to the energetic requirements of expressing the results of information processing in behavior. The strength of this proposal depends on further experimental investigation.

The data presented here are a subset of those presented in our recent report focused solely on changes in behavior, and the analyses of the current subsample reported here parallel those in that report (12). However, they differ from those we obtained in a study of the efficacy of a salt fortified with iron and iodine (5), in which we did observe significant changes in both low- and high-level cognitive functioning, along with changes in low-level perceptual functioning. We suggest that the difference—specifically, the lack of changes in behavioral measures of low-level attentional functioning in this and the companion report (12)—result in part from the relative challenge of the tasks presented to the 2 different populations. In particular, the present sample included university-aged women who, on the basis of their age, can be presumed to be at their life-span minimum for RTs (27, 28). In addition, the mean RTs in the tasks that assessed low-level cognitive functioning (including the ANT) are consistent with those reported for healthy college-aged participants in the US (17). This highlights the need to consider the relative difficulty of tasks used to assess the effects of nutritional status on cognitive functioning, as tasks that pose limited demands may not be sensitive to the presence of actual deficits. It was also the case that the 2 studies (5, 12) differed in iron dose [8.6 mg/d with 10% assumed bioavailability in (5), 5.9 mg/d with lower assumed bioavailability in the present effort and (12)].

The present report adds to the accumulating evidence regarding the effect of iron status on brain function, as measured by EEG. For example, Otero and colleagues (13, 14) have provided evidence that iron status affects working memory and ERPs indicative of mid- to high-level cognitive processing. Our findings that an ERP related to object-level representation [the N1, see (29)], and spectral power effects related to aspects of attentional control are consistent with those earlier reports. Taken together with the behavioral results, the EEG results suggest that changes in iron status are most likely to be observed in the components of complex cognitive performance (e.g., the instant-by-instant control of attention, word-by-word retrievals from memory in generating a spoken sentence) rather than in gross measures of cognitive status. Further, the consistent role of both SF and Hb in the mediation models suggests the potential need to concurrently measure the influence of iron-dependent neurotransmitter status and capacity for energy expenditure during cognitive performance.

The strengths of the present study include a principled selection of behavioral tasks on the basis of knowledge of the brain systems that are hypothesized to be affected by variations in systemic iron, instrumentation calibrated and sensitive enough to measure small but significant variations in speed of responding, and the inclusion of measures of brain dynamics to allow for statistical tests of the implicit causal relations linking blood, brain, and behavior. Weaknesses include floor effects on behavioral measures of low-level cognitive function that were picked up in part in the EEG measures, and a sample size that was smaller than desirable, but adequate to obtain statistical reliability. In sum, we believe that the results of the present study advance an understanding of how ID affects brain function and behavior, suggest important directions for future experimental work, and, in the context of findings with populations of different ages, invite further consideration of the implications of ID for brain health across the life-span.

## Supplementary Material

nxy265_Supplement_File

## Notes

Supported by a grant from HarvestPlus/IFPRI.

Author disclosures: MJW and JDH served as paid technical consultants for HarvestPlus/IFPRI. The funders of this study had no role in the study design, implementation, data collection, data analysis, data interpretation, or writing of the manuscript.

Supplemental Table 1 and Supplemental Figures 1 and 2 are available from the “Supplementary data” link in the online posting of the article and from the same link in the online table of contents at https://academic.oup.com/jn/.

Abbreviations used: ANT, attentional network task; BFB, biofortified bean; BL, baseline; CRT, cued recognition task; EEG, electroencephalography; EL, endline; ERP, event-related potential; GNG, go/no-go task; Hb, hemoglobin; ID, iron deficiency; RT, reaction time; SF, serum ferritin; SMS, Sternberg memory search task; SRT, simple reaction time task; sTfR, soluble transferrin receptor
