# Supplementary material for: Changes in Iron Status Are Related to Changes in Brain Activity and Behavior in Rwandan Female University Students: Results from a Randomized Controlled Efficacy Trial Involving Iron-Biofortified Beans
Source: J Nutr. 2019 Mar 30;149(4):687–97. doi: 10.1093/jn/nxy265 (PMC6461719; doi:10.1093/jn/nxy265)
Supplement: nxy265_Supplement_File [file nxy265_supplement_file.docx]

**Supplemental methods**

*EEG data pre-processing*

The acquired EEG data for each individual subject were prepared for statistical analyses in the following way. All pre-processing was done using EEGLAB, an open-source EEG analysis toolbox (23). First, the data were visually inspected and noisy or otherwise obviously problematic channels were identified and deleted. The data were then subjected to automated procedures for the detection of artifacts, including motion artifacts and blinks. The data were then filtered using a 1 to 90 Hz bandpass filter along with a 50 Hz notch filter. The data were segmented with respect to the onset of the task stimulus, retaining a 500 ms pre-stimulus baseline period and a 1,200 ms post-stimulus processing period. Next, an independent components analysis was performed, and components associated with artifacts and non-brain activity were identified and deleted automatically. Following this, the global field power for the data (24) was estimated, as the basis for identifying critical features in the event-related potentials. Three canonical ERP features were selected for each task: (a) the P1, a positive-going feature, occurring approximately 100 ms following the onset of the task stimulus, reflecting initial perceptual processing; (b) the N1, a negative-going feature, occurring between 150 and 250ms, reflecting object-level identification and processing; and (c) the P2, a positive-going feature occurring between 300 and 500ms following the onset of the task stimulus, reflecting semantic processing. In order to identify these features, the peaks in the global field power within these times windows was first identified. The timing of these peaks in the global field power was then used to select the electrode having the greatest amplitude within an 80 ms window centered on the time of the peak in the global field power. The peak amplitude and the time from the onset of the stimulus to this peak were then retained as variables for analysis. In addition, a Fourier analysis was performed on the segments, and normalized spectral power for the α- (8-15 Hz) and γ-bands (30-90 Hz) was obtained for each electrode.

*Procedural details: Behavioral tasks*

**Simple reaction time task (SRT).** The simple reaction time task provides an estimate of the simplest possible behavioral response to a visual stimulus, in that the task required the participant to press a button in response to the onset of the task stimulus. The task requires no discrimination or decision-making, has limited attentional and no mnemonic demands. The task stimulus (Supplemental Figure 1) was presented a total of 20 times. The interstimulus interval (ISI) varied on each trial for each participant and was determined using a uniform distribution running from between 750 and 1500 ms. The participant was instructed to press the “/” key on the computer keyboard as soon as she perceived the stimulus onset. Five practice trials preceded the test trials; feedback was provided only on the practice trials.

**Go/no-go task (GNG)**. The go/no-go task assesses simple sustained attention and response control. Stimuli were a horizontal and vertical white bar, centered on a black background, presented on screen. Stimuli were randomly, across both subjects and assessments, assigned to be either the go or the no-go stimulus. Subjects were instructed to press the / key with the index finger of their dominant hand on trials on which the go stimulus was presented, and to withhold a response on trials on which the no-go stimulus was presented.

Prior to beginning the task, subjects completed 10 practice trials, consisting of a random ordering of 2 go and 8 no-go trials, each preceded by a centrally presented fixation cross. The exposure duration for the fixation cross was randomly determined on each presentation by a value drawn from a uniform distribution over the interval from 400 to 700 ms. The stimulus was then presented for 300 ms. If the subject did not respond to the stimulus within 1700 ms, the sequence moved on to the next fixation and stimulus. The experimenter provided verbal feedback on trials on which the subject made either an error of commission or omission. Following the practice trials, the test trials were presented, consisting of a random ordering of 30 go trials and 120 no-go trials, using the timings that were used on the practice trials.

**Attentional network task (ANT).** The attentional network task is intended to probe three functions of attention: low-level attentional capture, mid-level spatial selective attention, and high-level control in the context of information that is nominally irrelevant to the performance of the task. The ANT was implemented according to the description in Fan et al. (1) and as such was run as a 5 (cue type: none, center, double, top, bottom) × 2 (target direction: left, right) × 3 (flanker type: neutral, congruent, incongruent) factorial design with all factors manipulated within-participants, and with six repetitions at each of the combinations of the factor levels. Order of presentation was determined randomly for each participant in each testing session.

Each trial of the task was initiated by the experimenter and began with a fixation cross presented in the center of the screen. The fixation duration was determined randomly on each trial using a value drawn from a uniform distribution over the interval running from 300 to 600 ms. The fixation cross was then replaced by a cue (an asterisk) in the appropriate location, with the cue being present for 200 ms. This was then replaced by a second display of the fixation cross for 200 ms, followed by the test stimulus—a left- or right-pointing arrow that was presented among a set of flanking elements, either above or below fixation (Supplemental Figure 1)—which was present for up to 1500 ms, or until the participant responded. Participants indicated their judgment of the direction of the target arrow using the index fingers of their left and right hands, pressing the “z” or “/” keys. The test trials were preceded by five practice trials (selected randomly for each participant in each session). The practice trials differed from the test trials only in that they were followed by feedback on the participant’s response.

**Sternberg memory search task (SMS).** The Sternberg memory search task measures the rate with which memory for very recent information can be searched, with the search rate differing systematically for old (previously encountered) rather than new (at test) information. Specifically, the search rates (ms/item) for new items are linearly related to the number of items that had to be remembered (potentially reflecting an exhaustive search), while the search rates for old items are much shallower (potentially reflecting a self-terminating search). In both cases, the slopes can be interpreted in terms of the efficiency with which a person can search memory for recent information.

The SMS was implemented as a 3 (set size: 1, 3, 6 items to be remembered) x 2 (test item: old, new) complete factorial design, with both factors manipulated within subjects and there being 10 replicates at each level of the design. Participants were instructed to press a key (either "z" or "/") under the index finger of their dominant hand if they judged a test item to be old, and to press the other key with the index finger of their non-dominant hand if they judged the test item to be new. Stimuli for this task were randomly-selected (with no repetition) ASCII graphics characters, presented as white on a black background (Supplemental Figure 1).

Each trial was initiated by the experimenter, and began with a centrally-presented fixation cross, with a duration determined randomly on each trial by a value drawn from a uniform distribution over the interval from 400 to 1000 ms. This was followed by the sequential presentation of the set of items (1, 3, or 6, randomly determined on each trial) to be remembered. There was a 2 sec blank interval following the presentation of the last item in the set, followed by the onset of the test item. No feedback was provided and there was a constant 1 s delay before the next trial could begin.

**Cued recognition task (CRT).** The cued recognition task is a variation on the classic recognition memory paradigm in which an individual is presented with a set of items to be remembered and is then tested on those items and an equal number of previously unseen items, and asked (for each test item) for a judgment as to whether the item was previously seen (an “old” item) or not (a “new” item). Critically, this task was included because of evidence suggesting that decrements in the amount of work that can be accomplished during recognition is correlated with the level of compromise to neural circuits—particularly those involving the hippocampus—that support recognition memory.

The CRT was implemented as a 2 (test item type: old, new) × 3 (number of cues at test: 2, 3, 4) factorial design, with all factors manipulated within participants. There were four trials at each possible combination of factors. Stimuli (Supplemental Figure 1) were a set of gray scale images, obtained from two sources, the first being a standardized and normed set of images created for use in recognition memory tasks and the second being a set of picture vocabulary images in use in a school in a nearby city. All images were judged to be easily identifiable, name-able, and culturally appropriate by local research staff.

The task was performed in two phases. The first was a study phase, in which participants were told that they would be seeing a set of pictures, and that the task was to study them in advance of a memory test that would follow. The complete set of study trials was initiated by the experimenter. The set of images was then presented, randomized for each participant in each session, at a rate of 3 s/image. The experimenter then gave the instructions for the test trials, which were initiated by the experimenter. Each trial consisted of the presentation of a test image (either an old or new item) with the appropriate number of cues (2, 3, or 4). The recognition cues were the number of equally-sized quadrants of the image that were visible. This was done by dividing the images into four 300 × 200 pixel quadrants. A random set of the appropriate number of quadrants (determined for each subject on each trial in each session) was covered by a black square. Participants were instructed to give a positive recognition judgment (”old”) using the index finger of their right hand on the “/” key of the computer keyboard, and a negative recognition judgment (”new”) using the index finger of their left hand on the “z” key. Participants were instructed to respond as quickly and as accurately as possible, and feedback was not provided.

Supplemental Table 1: Baseline and endline means (standard errors) for the blood, behavioral, and EEG variables for the comparison (CN) and biofortified (BFB) beans. The variable (Var) numbers (left-most column) refer to the variable number in Figure 2.

|  |  | CN (*N* = 28) | | | BFB (*N* = 27) | | |
| --- | --- | --- | --- | --- | --- | --- | --- |
| Var # | Task: Variable (units) | BL | EL | Δ | BL | EL | Δ |
|  | *Blood variables* |  |  |  |  |  |  |
| 1 | Hemoglobin (g/dL) | 12.35 (0.24) | 12.17 (0.22) | -0.18 (0.09) | 11.71 (0.32) | 12.12 (0.31) | 0.41 (0.14) |
| 2 | Ferritin (μg/L) | 9.52 (0.69) | 10.99 (0.86) | 1.79 (0.59) | 10.67 (1.35) | 16.02 (1.87) | 5.35 (1.45) |
| 3 | log10(Ferritin) | 0.95 (0.03) | 1.01 (0.03) | 0.07 (0.03) | 0.94 (0.06) | 1.13 (0.05) | 0.19 (0.04) |
| 4 | Transferrin receptor (mg/L) | 7.90 (0.76) | 8.21 (0.60) | 0.20 (0.35) | 8.19 (0.91) | 8.72 (0.90) | 0.54 (0.46) |
| 5 | Body iron (mg/kg) | -0.63 (0.43) | -0.44 (0.39) | 0.33 (0.25) | -0.83 (0.67) | 0.48 (0.62) | 1.31 (0.35) |
|  |  |  |  |  |  |  |  |
|  | *Behavioral variables* |  |  |  |  |  |  |
| 6 | SRT: median RT (ms) | 279 (12) | 300 (13) | 21 (8) | 271 (7) | 274 (7) | 3 (7) |
|  |  |  |  |  |  |  |  |
| 7 | GNG: median RT (ms) | 370 (10) | 357 (10) | -14 (7) | 382 (12) | 343 (9) | -39 (10) |
|  |  |  |  |  |  |  |  |
| 8 | ANT: alerting score (ms) | 38 (5) | 130 (6) | 91 (7) | 29 (7) | 126 (6) | 97 (9) |
| 9 | ANT: RT, 0 cues (ms) | 619 (12) | 574 (8) | -47 (10) | 630 (13) | 572 (9) | -58 (11) |
| 10 | ANT: RT, 2 cues (ms) | 580 (11) | 445 (7) | -138 (11) | 601 (14) | 446 (9) | -156 (11) |
| 11 | ANT: orienting score (ms) | 45 (5) | 163 (5) | 117 (7) | 46 (5) | 179 (6) | 132 (8) |
| 12 | ANT: RT, center cues (ms) | 602 (12) | 544 (7) | -60 (10) | 611 (14) | 549 (8) | -64 (12) |
| 13 | ANT: RT, spatial cues (ms) | 556 (10) | 382 (7) | -178 (9) | 565 (13) | 370 (8) | -196 (10) |
| 14 | ANT: conflict score (ms) | 174 (7) | 92 (5) | -85 (8) | 159 (6) | 90 (6) | -72 (6) |
| 15 | ANT: RT, consistent flankers | 603 (11) | 545 (7) | -59 (9) | 617 (14) | 543 (9) | -75 (11) |
| 16 | ANT: RT, inconsistent flankers | 776 (11) | 639 (8) | -141 (10) | 776 (15) | 602 (9) | -176 (12) |
|  |  |  |  |  |  |  |  |
| 17 | SMS: RT intercept, new items (ms) | 861 (52) | 855 (24) | -16 (51) | 943 (64) | 738 (21) | -195 (66) |
| 18 | SMS: RT intercept, old items (ms) | 796 (46) | 862 (25) | 51 (39) | 893 (64) | 708 (27) | -155 (53) |
| 19 | SMS: RT slope, new items (ms/item) | 47 (9) | 52 (4) | 4 (8) | 51 (10) | 34 (3) | -16 (11) |
| 20 | SMS: RT slope, old items (ms/item) | 60 (6) | 57 (6) | -3 (8) | 64 (10) | 32 (5) | -36 (11) |
|  |  |  |  |  |  |  |  |
| 21 | CRT: sensitivity, 4-cue trials (SD) | 2.57 (0.17) | 2.49 (0.05) | -0.07 (0.16) | 2.90 (0.20) | 3.46 (0.12) | 0.57 (0.18) |
| 22 | CRT: criterion, 4-cue trials (SD) | -0.13 (0.08) | 0.03 (0.06) | 0.16 (0.09) | -0.02 (0.07) | -0.24 (0.07) | -0.22 (0.09) |
| 23 | CRT: RT 4-cue trials, new items (ms) | 834 (42) | 839 (30) | 8 (35) | 836 (33) | 689 (13) | -147 (32) |
| 24 | CRT: RT 4 cue trials, old items (ms) | 771 (29) | 808 (26) | 25 (21) | 816 (30) | 720 (21) | -96 (31) |
| 25 | CRT: Percent change in capacity (%) | 49 (6) | 67 (3) | 18 (6) | 59 (7) | 131 (13) | 73 (14) |
|  |  |  |  |  |  |  |  |
|  | *EEG variables* |  |  |  |  |  |  |
| 26 | SRT: N1 amplitude, central (μV) | -3.95 (0.23) | -1.88 (0.33) | 1.92 (0.45) | -3.59 (0.37) | -4.59 (0.40) | -1.00 (0.70) |
| 27 | SRT: P2 amplitude, central (μV) | 1.37 (0.12) | 1.22 (0.05) | -0.15 (0.12) | 1.04 (0.10) | 1.49 (0.10) | 0.45 (0.17) |
| 28 | SRT: α-power, central (normalized) | 0.62 (0.01) | 0.61 (0.01) | -0.01 (0.01) | 0.61 (0.01) | 0.65 (0.01) | 0.04 (0.02) |
|  |  |  |  |  |  |  |  |
| 29 | GNG: N1 amplitude, central (μV) | -2.75 (0.30) | -3.74 (0.36) | 0.99 (0.32) | -2.10 (0.37) | -4.78 (0.53) | -2.68 (0.43) |
| 30 | GNG: P2 amplitude, central (μV) | 5.69 (0.45) | 5.55 (0.65) | -0.14 (0.59) | 6.30 (0.48) | 6.52 (0.83) | 0.22 (0.63) |
| 31 | GNG: α-power, central (normalized) | 0.64 ( 0.01) | 0.66 (0.01) | 0.02 (0.01) | 0.63 (0.01) | 0.69 (0.02) | 0.06 (0.02) |
|  |  |  |  |  |  |  |  |
| 32 | ANT: N1 amplitude, parietal, 0 cues (μV) | -3.74 (0.34) | -3.73 (0.28) | 0.01 (0.32) | -4.33 (0.44) | -4.74 (0.40) | -0.41 (0.42) |
| 33 | ANT: N1 amplitude, occipital, 2 cues (μV) | -1.53 (0.40) | -2.27 (0.38) | -0.74 (0.35) | -1.28 (0.43) | -3.87 (0.44) | -2.59 (0.37) |
| 34 | ANT: N1 amplitude, parietal, central cues (μV) | -3.94 (0.30) | -3.68 (0.30) | 0.26 (0.36) | -4.06 (0.27) | -4.50 (0.35) | -0.44 (0.37) |
| 35 | ANT: N1 amplitude, parietal, spatial cues (μV) | -2.42 (0.28) | -2.37 (0.21) | 0.05 (0.22) | -2.82 (0.32) | -3.47 (0.25) | -0.65 (0.33) |
| 36 | ANT: N1 amplitude, occipital, consistent flankers (μV) | -1.79 (0.33) | -2.02 (0.31) | -0.23 (0.25) | -1.77 (0.33) | -4.24 (0.42) | -2.47 (0.39) |
| 37 | ANT: N1 amplitude, occipital, inconsistent flankers (μV) | -1.63 (0.28) | -2.04 (0.34) | -0.41 (0.31) | -1.75 (0.34) | -4.38 (0.32) | -2.63 (0.32) |
| 38 | ANT: α-power, central, 2 cues (normalized) | 0.68 (0.01) | 0.70 (0.01) | 0.02 (0.02) | 0.67 (0.01) | 0.72 (0.01) | 0.05 (0.01) |
| 39 | ANT: α-power, central, central cues (normalized) | 0.68 (0.01) | 0.70 (0.01) | 0.02 (0.01) | 0.68 (0.01) | 0.73 (0.01) | 0.05 (0.01) |
| 40 | ANT: α-power, central, spatial cues (normalized) | 0.68 (0.01) | 0.69 (0.01) | 0.01 (0.01) | 0.68 (0.01) | 0.72 (0.01) | 0.04 (0.01) |
| 41 | ANT: a-power, central, inconsistent flankers (normalized) | 0.65 (0.01) | 0.66 (0.01) | 0.01 (0.01) | 0.65 (0.01) | 0.71 (0.01) | 0.06 (0.01) |
|  |  |  |  |  |  |  |  |
| 42 | SMS: N1 amplitude, central, new items, set size 1 (μV) | -1.87 (0.15) | -1.82 (0.14) | -0.05 (0.21) | -2.14 (0.49) | -4.64 (0.14) | -2.50 (0.50) |
| 43 | SMS: N1 amplitude, central, old items, set size 1 (μV) | -2.49 (0.25) | -1.94 (0.18) | 0.50 (0.32) | -2.54 (0.55) | -4.95 (0.22) | -2.41 (0.53) |
| 44 | SMS: N1 amplitude, central, new items, set size 3 (μV) | -2.13 (0.20) | -1.94 (0.20 | 0.19 (0.30) | -2.17 (0.31) | -4.92 (0.23) | -2.75 (0.34) |
| 45 | SMS: N1 amplitude, central, old items, set size 3 (μV) | -2.35 (0.20) | -1.84 (0.17) | 0.51 (0.23) | -2.47 (0.29) | -4.75 (0.14) | -2.28 (0.29) |
| 46 | SMS: N1 amplitude, central, new items, set size 6 (μV) | -1.88 (0.18) | -1.68 (0.16) | 0.20 (0.24) | -2.09 (0.29) | -4.54 (0.14) | -2.45 (0.23) |
| 47 | SMS: N1 amplitude, central, old items, set size 6 (μV) | -2.45 (0.18) | -2.41 (0.35) | 0.04 (0.39) | -2.81 (0.39) | -5.11 (0.31) | -2.30 (0.50) |
| 48 | SMS: γ-power, central, old items, set size 1 | 0.67 (0.01) | 0.67 (0.01) | 0.00 (0.01) | 0.67 (0.01) | 0.70 (0.01) | 0.03 (0.01) |
| 49 | SMS: γ-power, central, old items, set size 3 | 0.44 (0.01) | 0.42 (0.01) | -0.02 (0.01) | 0.43 (0.01) | 0.45 (0.01) | 0.02 (0.01) |
| 50 | SMS: γ-power, central, old items, set size 6 | 0.66 (0.01) | 0.66 (0.01) | 0.00 (0.01) | 0.66 (0.01) | 0.69 (0.01) | 0.03 (0.01) |
|  |  |  |  |  |  |  |  |
| 51 | CRT: N1 amplitude, central, new items, 4 cues (μV) | -4.42 (0.47) | -2.90 (0.28) | 1.52 (0.42) | -4.28 (0.42) | -5.48 (0.32) | -1.20 (0.28) |
| 52 | CRT: N1 amplitude, central, old items, 4 cues (μV) | -3.62 (0.39) | -2.87 (0.33) | 0.75 (0.52) | -3.92 (0.57) | -5.59 (0.32) | -1.67 (0.59) |

Alphabetized list of abbreviations: BFB, biofortified beans; BL, baseline; CN, comparison beans; Δ, change, EL – BL; EL, endline; ms, milliseconds; RT, reaction time

| *Task* | *Example stimulus* | *Dimensions (deg visual angle)* |
| --- | --- | --- |
| Simple reaction time (SRT) | 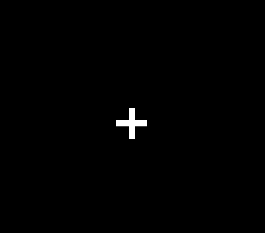 [ ]  a  b | a = 1.06  b = 1.74 |
| Go/No-Go (GNG) | a  b | a = 0.10  b = 1.20 |
| Attentional network (ANT) | 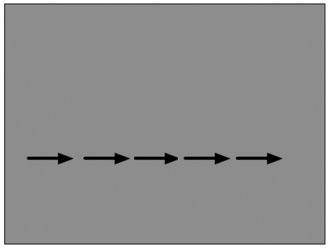 +  a  b | a = 1.10  b = 0.80 |
| Sternberg memory search (SMS) | 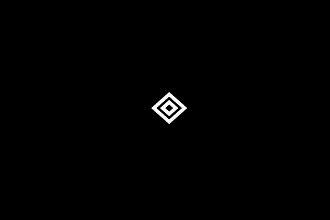 a  b | a = 1.06  b = 1.21 |
| Cued recognition (CRT) | 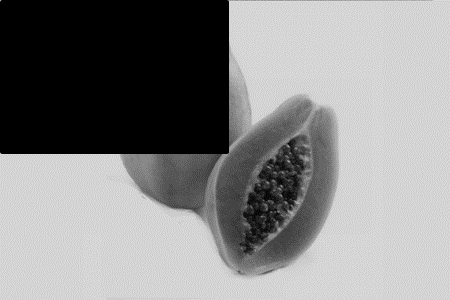 a  b | a = 12.09  b = 8.29 |

Supplemental Figure 1: Example stimuli and their dimensions, five cognitive tasks. Dimensions are given in degrees (deg) of visual angle at an unconstrained viewing distance of 70 cm.

Supplemental Figure 2: Schematic representation of the time-course for each of the event-related potential (ERP) features.
